# Cholesterol Depletion with U18666A and Methyl-β Cyclodextrin Increased Small Molecule Permeability Across Brain Microvascular Endothelial Cells

**DOI:** 10.1007/s10439-025-03841-9

**Published:** 2025-09-17

**Authors:** Bilal Moiz, Viviana Alpizar Vargas, Ken D. Brandon, Gurneet Sangha, Callie Weber, Andrew Li, Tristan Pepper, Matthew Walls, Anthony Qin, Sara Hart, Cristin Davidson, Kimberly Stroka, Forbes D. Porter, Alisa Morss Clyne

**Affiliations:** 1 University of Maryland, College Park, MD 20742 USA; 2 Eunice Kennedy Shriver National Institute of Child Health and Human Development, National Institutes of Health, Bethesda, MD 20892 USA

**Keywords:** Blood–brain barrier, Niemann-Pick disease, Tight junction, Cholesterol, iPSC-BMEC, Brain endothelial

## Abstract

Cholesterol is a vital component of the cell membrane and plays an essential role in mediating integral membrane protein function. Altered cholesterol regulation has been implicated in neurological diseases that are associated with blood–brain barrier breakdown. However, the role of brain barrier function in inherited disorders of cholesterol metabolism, such as Niemann-Pick disease C1 (NP-C1), remains unclear. In this study, we determined how cholesterol depletion with U18666A, a chemical inhibitor of NPC1 protein, as well as with the cholesterol-depleting agent methyl-β cyclodextrin (MβCD), impacted brain endothelial cell barrier function. We hypothesized that cholesterol depletion would decrease barrier integrity by disrupting tight junction protein continuity. To test this hypothesis, we differentiated human-induced pluripotent stem cells into brain microvascular endothelial cells (hiBMECs). We then assessed barrier integrity by quantifying trans-endothelial electrical resistance (TEER), small fluorescent molecule permeability, and tight junction continuity and protein levels. We now show that U18666A-treated hiBMECs demonstrated a 75% decrease in TEER and 9-fold increase in sodium fluorescein permeability. Similar trends were observed for hiBMECs treated with MβCD, which showed significantly lowered TEER (93% decrease) and increased sodium fluorescein permeability (20-fold higher). We also observed decreased continuity of the tight junction proteins occludin (13% lower) and claudin-5 (8% lower) as well as a 53% decrease in claudin-5 protein with U18666A treatment. Co-treating U18666A-treated hiBMECs with hydroxypropyl-β cyclodextrin (HPβCD), which releases lysosomal cholesterol, prevented these changes. Together, our results demonstrate that cholesterol is vital for hiBMEC barrier function and tight junction continuity. This study highlights the potential of therapeutics targeted to brain endothelium in NP-C1 and other cholesterol metabolism disorders.

**Supplementary Information:**

The online version contains supplementary material available at 10.1007/s10439-025-03841-9.

## Introduction

The blood–brain barrier (BBB) is critical for healthy neurological function, as it protects the brain from inflammatory and neurotoxic elements found in the blood [1]. The BBB is primarily formed by brain microvascular endothelial cells (BMECs) bound together by tight junction (TJ) proteins, including zonula occludens-1 (ZO-1), claudin, and occludin. Together, these proteins form a complex which seals adjacent cells together and eliminates paracellular gaps. As a result, only ions and small nonpolar molecules can diffuse between cells while larger compounds must instead transverse through selective transcellular transport pathways. BBB breakdown and dysfunction secondary to decreased tight junction continuity can allow toxic substances to infiltrate the brain and potentially contribute to or even precipitate neurological decline.

Notably, BBB dysfunction, including increased permeability, has been implicated in several neurological diseases, including Alzheimer’s disease, Parkinson’s disease, stroke, and multiple sclerosis [2, 3]. However, our current understanding of BBB dysfunction in rare inherited neurological disorders such as Niemann-Pick Disease Type C1 (NP-C1) remains limited. NP-C1 is an autosomal recessive disease typically caused by pathological variants in *NPC1*, which encodes an intracellular cholesterol transport protein that facilitates cholesterol movement through the late endosome/lysosome to the plasma membrane and other organelles [4]. On a cellular level, NP-C1 is defined by endolysosomal cholesterol and glycosphingolipid accumulation, with subsequent cholesterol depletion from the plasma membrane caveolae, endoplasmic reticulum, and golgi apparatus [5–8]. In the classic form of this disease, patients present with childhood onset of neurodegeneration, leading to cerebellar ataxia, loss of motor skills, intellectual disability, seizures, and ultimately early death [9].

Plasma membrane cholesterol alterations have previously been shown to disrupt barrier function. For example, cholesterol depletion with methyl-β cyclodextrin (MβCD) increased barrier permeability in the Caco-2 intestinal cell line by displacing claudins from tight junction-associated lipid rafts [10, 11]. Knockout of the adherens junction protein α-catenin, which depletes cholesterol from the plasma membrane, as well as MβCD treatment led to claudin loss from cell–cell junctions in mouse epithelial cells. Addition of exogenous cholesterol reversed this effect [12]. MβCD also decreased claudin-5 and increased BBB permeability in rats [13]. However, most studies investigating the effect of plasma membrane cholesterol depletion on barrier function were conducted in epithelial cells rather than BMECs and in rodent rather than human models. Accordingly, there is a need to examine the effect of plasma membrane cholesterol depletion on barrier function in human BMECs. An improved understanding of BBB changes with diminished plasma membrane cholesterol could inform novel therapeutic and preventative strategies in NP-C1, as well as other neurological disorders with altered plasma membrane cholesterol.

Plasma membrane cholesterol depletion has also been shown to impact barrier integrity by altering metabolism. Cholesterol is connected to both glycolysis and the mitochondrial TCA cycle through metabolic intermediates, substrates, and cofactors, particularly acetyl-CoA and NAPDH [14–16]. We previously demonstrated that inhibiting NPC1 in human-induced pluripotent stem cell-derived BMECs (hiBMECs) decreased mitochondrial metabolism while increasing glycolytic metabolism [17]. Decreased mitochondrial metabolism may impact barrier function through ATP, since tight junctions undergo dynamically remodeling in response to environmental stimuli in an energy-dependent manner [18]. Inhibition of mitochondrial ATP in Caco-2 epithelial cells led to a dose-dependent increase in barrier permeability [19]. Enhanced glycolysis has also been shown to be a key mechanism for decreasing endothelial cell barrier integrity, for example in response to histidine [20]. These results, along with our previous characterization of metabolic changes in U18666A-treated hiBMECs, suggest a connection among plasma membrane cholesterol depletion, metabolism, and barrier function.

In this study, we hypothesized that decreasing hiBMEC cholesterol would decrease barrier integrity by disrupting tight junctions. To test our hypothesis, we used both U18666A, a pharmacological NPC1 inhibitor [21], as well as the cholesterol-depleting agent MβCD to understand how decreased cholesterol impacts hiBMEC barrier function. We examined permeability changes through tight junction continuity analysis and size-selective permeability tracer assays. We then tested whether co-treating U18666A-treated hiBMECs with hydroxypropyl-β-cyclodextrin (HPβCD), a potential therapeutic compound that releases sequestered lysosomal cholesterol, could prevent changes in barrier function. Overall, this work emphasizes the potential of BBB dysfunction in cholesterol-altering diseases such as NP-C1 and highlights BMECs as potential therapeutic targets in NP-C1 and other conditions of altered cholesterol.

## Methods and Materials

### Cell Culture, Differentiation, and NPC1 Inhibition

IMR90-induced pluripotent stem cells [22] (IMR90-iPSCs; WiCell) were obtained and used following protocols approved by the Institutional Biosafety Committee at the University of Maryland, College Park. IMR90-iPSCs were differentiated into BMEC-like cells (hiBMECs) using previously established protocols [23, 24]. iPSCs were cultured on Matrigel-coated (Corning, 354230) plates in mTeSR-Plus medium (STEMCELL Technologies, 100-0276). Upon reaching 70% confluence, IMR90-iPSCs were detached using Accutase (ThermoFisher, A1110501), centrifuged at 1000 rpm for five minutes, resuspended in mTeSR-Plus medium containing 10 µM Y-27632 (ROCK inhibitor; Tocris, 1254), and then seeded at ~ 21,000 cells/cm^2^ on Matrigel-coated six-well plates. The next day, the medium was changed to E6 (STEMCELL Technologies, 05946) and replaced daily for the next four days. The medium was then changed to either human endothelial serum free media (hESFM; ThermoFisher, 11111044) or neurobasal medium (ThermoFisher, 21103049) supplemented with 2% B27 (ThermoFisher, 17504001), 20 ng/mL basic fibroblast growth factor (bFGF; Peprotech, 100-18B), and 10 µM retinoic acid (RA; Millipore Sigma, R2625-50MG). On day 6, the cells were detached with Accutase and then subcultured onto extracellular matrix (ECM) coated 0.4 μm pore Transwell filters (Corning, 3460), well plates, glass cover slips (confocal microscopy), or #1.5 coverglass black walled 96-well plates (Cellviz, P96-1.5P; high-throughput imaging). The ECM consisted of 50% water, 40% collagen IV (Millipore Sigma, C7521), and 10% fibronectin (Millipore Sigma, F2006).

For the majority of experiments, confluent hiBMECs were treated with hESFM supplemented with 2% B27 containing U18666A (Sigma-Aldrich, U3633), an NPC1 inhibitor that blocks free cholesterol from leaving the late endosome; methyl-β-cyclodextrin (MβCD; Sigma-Aldrich, C4555), which transiently removes cholesterol from the plasma membrane; 2-hydroxypropyl-β-cyclodextrin (HPβCD; Roquette, 128446), which releases sequesters cholesterol from the lysosome; or water-soluble cholesterol (CHOL; Sigma-Aldrich, C4951) [25]. For experiments with 2-deoxy-D-glucose (2-DG, ThermoFisher, 111980050), hiBMECs were treated for 48 h with 2-DG either with or without 10 μM U18666A.

### Cholesterol Quantity and Localization

Cholesterol quantity and localization were determined via Amplex Red and perfringolysin-O (PFO), respectively. For total cholesterol quantity, an Amplex Red Cholesterol Assay Kit (ThermoFisher) was used as per manufacturer protocol. Cells were lysed with 1x Reaction Buffer and homogenized on ice for 10 seconds. Homogenized samples were added to a 96-well plate with Amplex Red working solution and incubated at 37 °C for 30 minutes. Fluorescence was read using a Tecan Spark Multimode Microplate Reader at 530–590 nM excitation-emission. For protein quantification, 10 µL sample lysate was mixed with bicinchoninic acid (BCA) reagent (ThermoFisher, 23225), and absorbance was quantified at 562 nm with a Tecan Spark Multimode Plate Reader. Cholesterol concentration was then normalized to protein.

To visualize cholesterol localization, hiBMECs were seeded on an ECM-coated glass bottom 96-well plate. Following treatment, hiBMECs were washed with phosphate buffered saline (PBS), fixed with 4% paraformaldehyde for 15 minutes at room temperature, and then incubated with 5% goat serum containing 2 µg/mL Alexa Fluor 488-conjugated PFO (PFO-488; a generous gift from Forbes Porter), a pore-forming cytolysin that binds to free cholesterol [26, 27]. Cell nuclei were stained with Hoescht (1:2000, ThermoFisher, 33342), washed with PBS, and then imaged with a Nikon Eclipse T2i microscope in confocal mode (60X oil objective). Cholesterol intensity was quantified in Nikon Eclipse Software suite by dividing the sum of GFP intensity by the number of nuclei for each image. Three images were taken per well and averaged to obtain the mean value per well.

### RT-PCR

RNA was isolated from hiBMECs using an RNEasy Mini Kit (Qiagen), quantified on a Nanodrop 2000c (ThermoFisher), and converted to cDNA using a High-Capacity cDNA Reverse Transcription kit (ThermoFisher) and a ProFlex Thermal Cycler (ThermoFisher). qPCR was performed using a QuantStudio 7 Flex qPCR System (ThermoFisher), with a primer for CLDN5 (Hs00533949_s1). RPLP0, a ribosomal protein, was used as the housekeeper gene (Hs00420895_gH; all from ThermoFisher). RT-PCR data were analyzed using the comparative CT method.

### Transendothelial resistance (TEER) and permeability assays

Transendothelial resistance (TEER) was measured in hiBMECs on 0.4 µm pore Transwell inserts in triplicate using STX4-Plus electrodes and the Epithelial Volt/Ohm Meter 3 (EVOM3; World Precision Instruments). Resistance values were corrected by subtracting the resistance of a cell-free Transwell insert. TEER values are reported as Ohms x cm^2^, for which the resistance value was multiplied by the Transwell insert area (1.12 cm^2^ for 12-well; 0.33 cm^2^ for 24-well).

For permeability experiments, hiBMEC media were replaced with fresh hESFM with 2% B27 and either 10 µM sodium fluorescein (Sigma-Aldrich, F6377) or 200 μg/mL 4 kDa (Sigma-Aldrich, 46944), 10 kDa (Thermofisher Scientific, D1863), or 70 kDa dextran (Sigma-Aldrich, 53471). Every 15 minutes, 50 μL of basolateral media was removed, placed in a black bottom 96-well plate, and replaced with 50 μL fresh media. After one hour, 5 μL of apical media was collected, placed in a black bottom 96-well plate, and diluted 1:10 with 45 μL hESFM + 2% B27 media. Fluorescence values were then quantified with a Tecan Spark Multimode Plate Reader, using the appropriate excitation and emission settings. Intensity values were corrected for background and signal loss. Apical media fluorescence intensities were multiplied by 10 to account for dilution.

Clearance volume and permeability coefficient values were calculated as previously described [28]. Briefly, the basolateral fluorescence intensity values were corrected for the volume removed at each time point. Clearance volume (μL) was then calculated by multiplying the corrected fluorescence intensity values by the total basolateral media volume (μL) and normalizing to apical media fluorescence intensity as

$$\text{Clearance} \text{volume} =\frac{\text{Basolateral} \text{volume}\times \text{Basolateral} \text{fluorescence} \text{intensity}}{\text{Apical} \text{fluorescence} \text{intensity}}$$

The clearance rate C_r_, defined as the change in clearance volume over time ($\frac{\text{d}C}{\text{d}T}$), was normalized to the clearance volume of a cell-free sample and used to calculate the inverse permeability coefficient ($\frac{1}{{P}_{\text{coeff}}}$) as

$$\text{Inverse} \text{permeability} \left(\frac{1}{{P}_{\text{coeff}}}\right)=\frac{1}{{C}_{r, \text{sample}}}-\frac{1}{{C}_{r, \text{cell}-\text{free}}}$$

The permeability coefficient was then normalized to the Transwell insert area.

### Cell–Cell Junction Visualization and Quantification

To visualize tight junctions, hiBMECs were fixed in either ice-cold methanol or 4% paraformaldehyde supplemented with 0.2% Triton X-100 (Alfa Aesar, A16046) to permeabilize cells for 20 minutes. Samples were blocked with 5% donkey serum (Sigma-Aldrich, S30) in PBS for one hour. Cells were then incubated with primary antibodies (Table 1) overnight at 4 °C. After thorough washing, cells were labeled with the appropriate secondary antibodies (Table 1). Samples were imaged on a Nikon 2Ti Eclipse microscope with a 60X oil (for tight junction morphology analysis) objective in confocal mode. Each image was processed in NIS elements using the rolling ball module to correct background. A maximum intensity projection image was created, and the compressed images were exported as TIF files for further analysis.

**Table 1:** Immunofluorescence and western blot antibodies

Target protein	Company, product #	Immunofluorescence: fixation, dilution, secondary antibody	Western blot: membrane, dilution, secondary antibody
Claudin-5	ThermoFisher, 4C3C2	Methanol, 1:50 Donkey-anti-Mouse 488 (ThermoFisher, A-21202)	PVDF, 1:1000 Anti-Mouse (Promega, W4021)
ZO-1	Cell signaling, 13663S	Methanol, 1:50 Donkey-Anti-Rabbit 488 (ThermoFisher, A-21206)	Nitrocellulose, 1:500 Anti-Rabbit IgG (Promega, W4011)
Occludin	Cell signaling, 13663S	Methanol, 1:50 Donkey-Anti-Rabbit 488 (ThermoFisher, A-21206)	PVDF, 1:1000 Anti-Rabbit IgG (Promega, W4011)
Occludin	ThermoFisher, 33-1500	Methanol, 1:50 Donkey-Anti-Mouse 594 (ThermoFisher, A-21203)	N/A
VE-Cadherin	R&D systems, AF938	N/A	PVDF, 1:1000 Anti-Goat IgG (ThermoFisher Scientific R-21459)
p-VE Cadherin	Sigma-Aldrich, ABT1760	N/A	PVDF, 1:1000 Anti-Mouse (Promega, W4021)

Tight junction continuity was analyzed using the Junction Analyzer Program (JAnaP; https://github.com/StrokaLab/JAnaP) [29]. We delineated the perimeters of randomly selected cells by first defining points on the cell edges (waypoints). JAnaP then connected the waypoints by creating a path between them that followed the maximum fluorescence intensity. The paths were then automatically stitched together to create the full cell perimeter. Each pixel along the cell perimeter was characterized as having a junction or no junction based on a fluorescent intensity threshold (set at 15 across all images). The cell perimeter sections were then categorized as having continuous junctions if > 15 adjacent pixels along the cell perimeter were above the defined fluorescent intensity threshold [29]. The percent continuity was finally calculated by summing the lengths of all continuous segments and dividing that by the total cell perimeter. At least 5 cells were randomly selected and traced per image.

### Western Blot

To analyze intracellular proteins, cells were lysed in RIPA buffer (ThermoFisher, 89901) supplemented with Halt Protease and Phosphatase Inhibitor (ThermoFisher, PI78440). Protein concentration was quantified via BCA assay. Normalized protein samples, sample buffer (ThermoFisher, NP0008), and reducing agent (ThermoFisher, NP0009) were combined, heated at 70 °C for 5 min, and then separated using 4–12% Bis-Tris gels (ThermoFisher, NP0323). Proteins were transferred to PVDF (ThermoFisher, IB24001) or nitrocellulose (ThermoFisher, IB23001) membranes using an iBlot2 (ThermoFisher, IBL21001). Membranes were then blocked for 1 hour in 5% bovine serum albumin in PBS containing 0.5% Tween 20 (ThermoFisher, 85113) to reduce non-specific binding. Membranes were incubated in primary antibodies overnight at 4 °C (Table 1), followed by 2 h in the respective horseradish peroxidase conjugated-secondary antibody at room temperature (Table 1). Protein bands were imaged using an Alpha Innotech Fluorchem Imager (Protein Simple). Band intensities were analyzed using AlphaView software.

### Statistical Analysis

Statistics were analyzed using Prism 10.3.1 (GraphPad). Non-parametric Mann–Whitney tests were used to compare two unpaired datasets, except for JAnaP data, which was analyzed with a Kolmogorov–Smirnov test. One-way Kruskal–Wallis non-parametric test was used to compare multiple groups, followed by *post hoc* Dunn’s multiple comparison test. Data for JAnaP analysis were considered statistically significant if *p* < 0.0001. For all other experiments, data were considered statistically significant if *p* < 0.05.

## Results

### U18666A and MβCD Reduced hiBMEC Cholesterol and Impaired Barrier Integrity

**Fig. 1:**
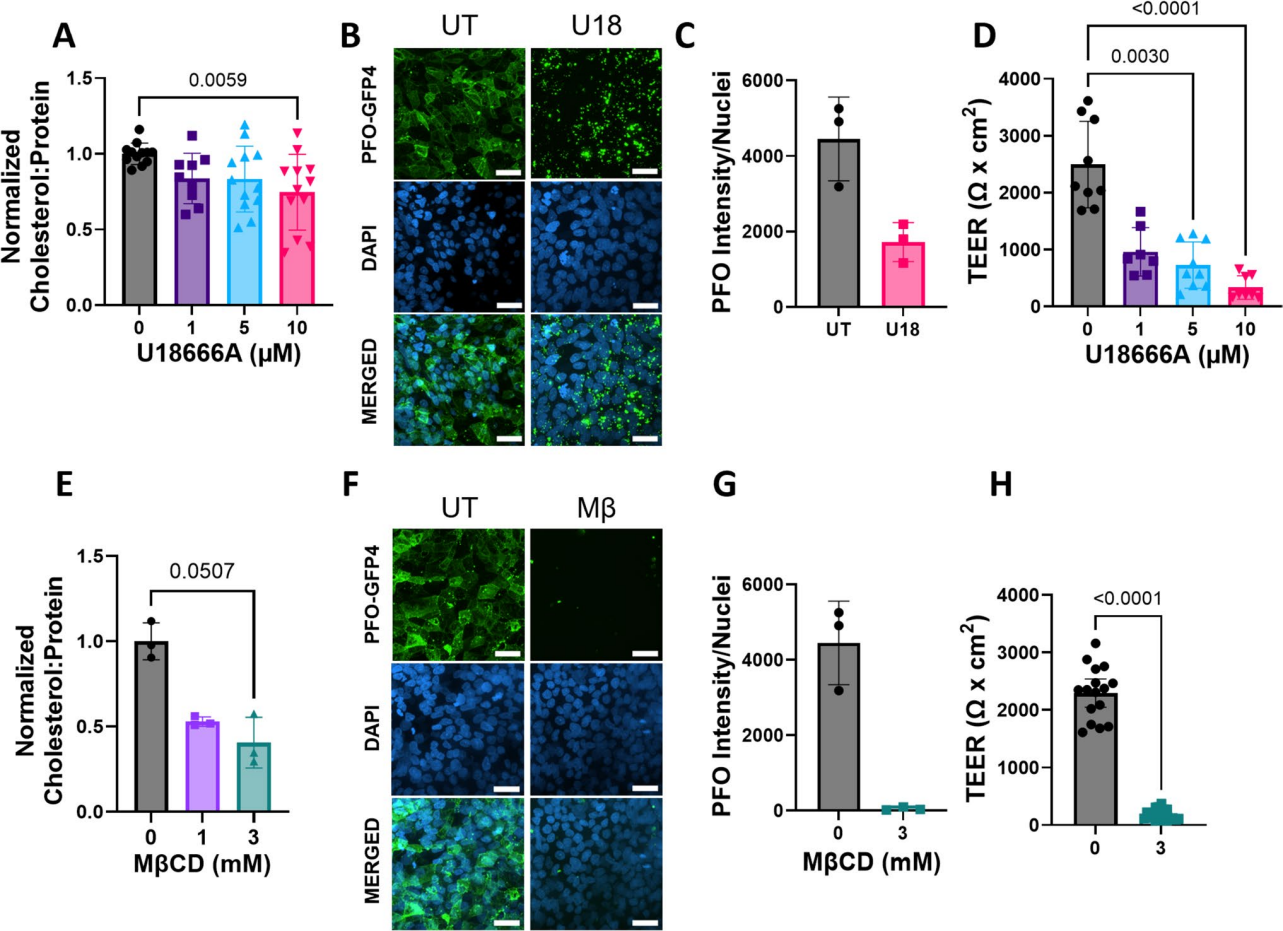
U18666A and MβCD treatment reduced hiBMEC cholesterol levels and barrier integrity. **A** Cholesterol content from hiBMECs treated with 0, 1, 5, or 10 μM U18666A for 48 h and analyzed with an Amplex Red assay. **B** Representative images and (**C**) quantification of hiBMECs treated with 10 μM U18666A for 48 h, then fixed and labeled with PFO-488 (green) and DAPI (blue) to visualize cholesterol. Scale bar = 25 μM (*n* = 3, one experiment). **D** hiBMEC TEER after 48 h of 0, 1, 5, or 10 μM U18666A treatment (*n* = 9–12 from 3 independent experiments). **E** Cholesterol content from hiBMECs treated with 0, 1, or 3 mM MβCD. Cholesterol levels were measured with an Amplex Red assay (*n* = 3, one experiment). **F** Representative images and (F) quantification of hiBMECs treated with 3 mM MβCD for 3 h then fixed and labeled with PFO-488 (green) and DAPI (blue) to visualize membrane cholesterol. Scale bar = 25 μM (*n* = 3, one experiment). **H** hiBMEC TEER measurements after 3 h of treatment with 0 or 3 mM MβCD (*n* = 16–17 samples). All data shown as mean + /− sd. Significance determined with Kruskal–Wallis non-parametric test followed by Dunn’s multiple comparison test when comparing multiple groups or Mann–Whitney test when comparing two groups.

We first determined the dose and time at which U18666A or MβCD reduced hiBMEC cholesterol. Total cellular cholesterol, as measured by Amplex Red, decreased in a dose-dependent manner in U18666A-treated hiBMECs, with a nearly 25% decrease in hiBMECs treated with 10 μM U18666A for 48 h (*p* = 0.0059; Fig. 1A). We further showed that U18666A decreased membrane cholesterol using PFO-488, which associates with free cell membrane cholesterol. PFO-488 fluorescent intensity was more than 50% lower in U18666A-treated hiBMEC, and cholesterol was redistributed into perinuclear puncta (Fig. 1B, C). The PFO assay may have shown a larger decrease in cholesterol with U18666A treatment since PFO binds primarily to free membrane cholesterol and was imaged at the cell surface, whereas the Amplex Red assay quantifies total free cholesterol and cholesterol esters throughout the cell. These data agree with our previous work, in which we showed that treating hiBMECs with 10 μM U18666A for 48 h reduced overall cholesterol and redistributed cholesterol from the membranes to the lysosome without any loss of cell viability [17].

We then assessed the impact of U18666A on hiBMEC barrier integrity by measuring TEER. TEER decreased with increasing U18666A concentration (Figure 1D). After 48 h of 10 μM U18666A, hiBMEC TEER was 88% lower than untreated cells (*p* < 0.0001). A statistically significant decrease (*p* = 0.0030) was also observed with 5 μM U18666A. NPC1 inhibition can cause lysosomal cholesterol accumulation in addition to membrane cholesterol depletion. To test whether impaired hiBMEC barrier function with U18666A could be explained by cholesterol depletion independent of lysosomal cholesterol accumulation, we treated hiBMECs with the cholesterol-depleting agent MβCD. Treatment with 3 mM MβCD for 3 h reduced hiBMEC cholesterol levels by 60% by Amplex Red assay (*p* = 0.0507; Figure 1E). PFO-488 showed a nearly complete loss of membrane cholesterol with 3 mM MβCD at 3 h (Fig. 1F, G), with membrane cholesterol recovery by 24 h (data not shown). Treatment with 3 mM MβCD for 3 h reduced hiBMEC TEER to 8% of starting values (*p* < 0.0001) (Fig. 1H). These data show that membrane cholesterol depletion impaired hiBMEC barrier function.

### hiBMEC Cholesterol Depletion Increased Small Molecule Permeability

**Fig. 2:**
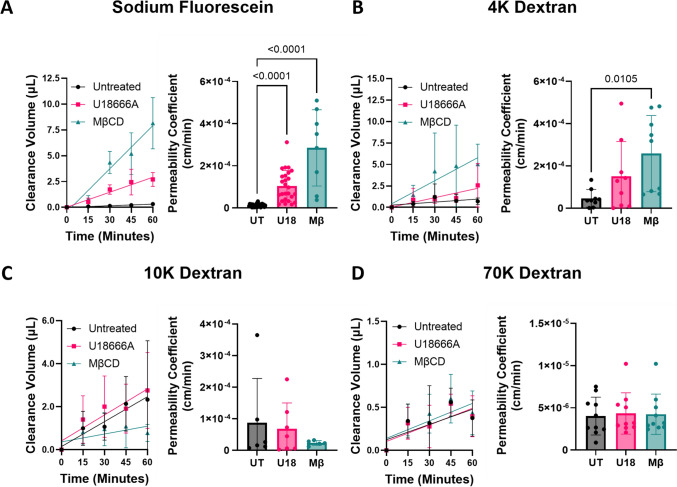
NPC1 inhibition increased hiBMEC permeability to small molecules (< 10 kDA) only. hiBMEC were treated with solvent control (UT, 48 h), U18666A (U18, 48 h), or MβCD (Mβ, 3 h). Clearance volume vs. time and permeability coefficients for **A** sodium fluorescein (MW = 376.7 Da, *n* = 8–26 from 4 independent experiments), **B** 4 kDa dextran (*n* = 9, from 3 independent experiments) **C** 10 kDa dextran (*n* = 5–7 from 3 independent experiments), and (D) 70 kDa dextran (*n* = 10 from 3 independent experiments). Data shown are mean + /− SD. Statistical significance determined with Kruskal–Wallis non-parametric test followed by Dunn’s multiple comparison test.

**Fig. 3:**
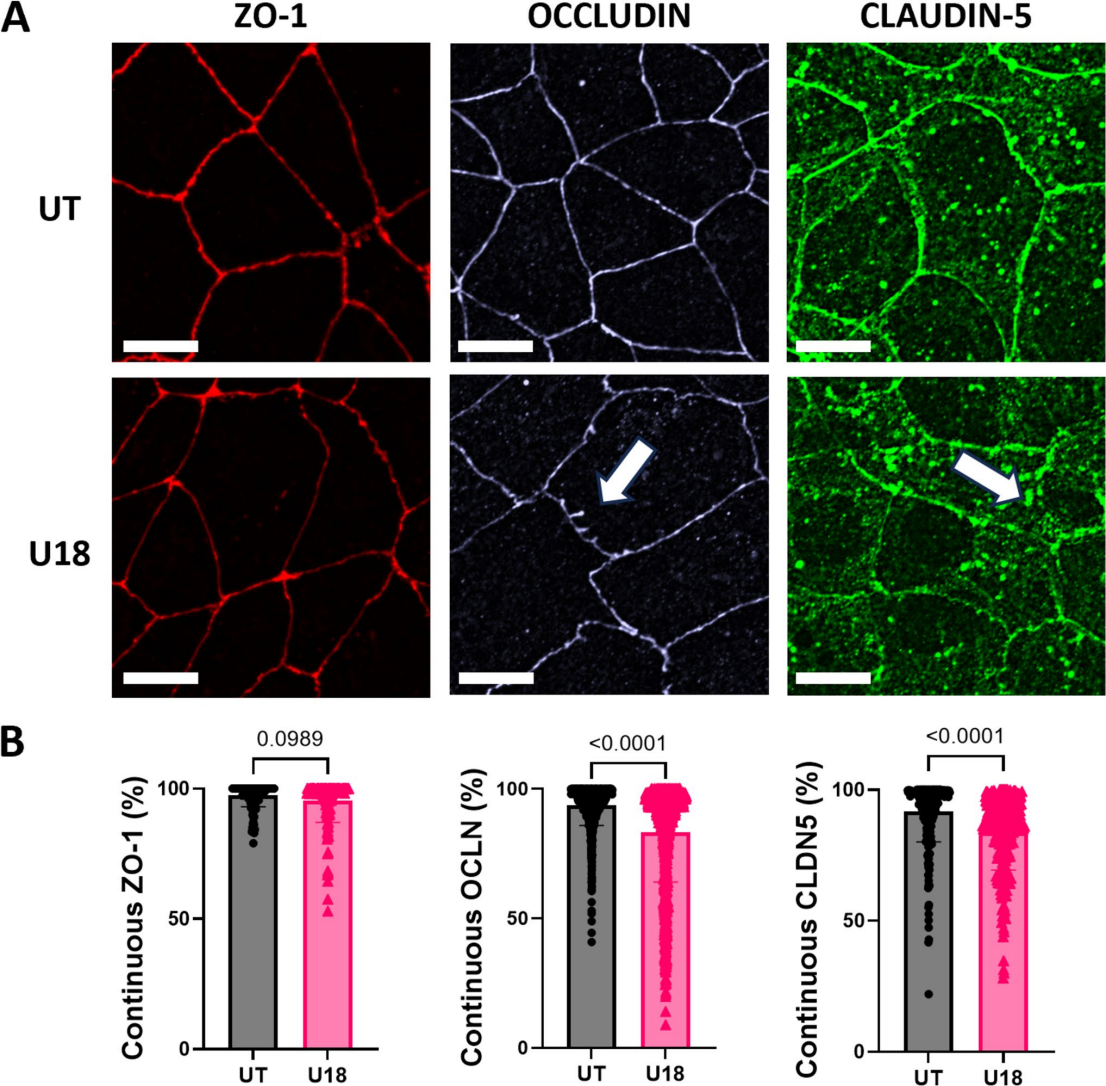
Cholesterol depletion decreased occludin and claudin-5 continuity without affecting ZO-1. **A** Representative images of ZO-1 (red), occludin (light blue), and claudin-5 (green). White arrows indicate discontinuous tight junctions. Scale bar = 25 μM. **B** Percent of continuous tight junctions for ZO-1 (*n* = 39–98 cells), occludin (OCLN, *n* = 726–827 cells), and claudin-5 (CLDN, *n* = 119–221 cells) as quantified by JAnaP. Data shown are mean + /− SD. Statistical significance determined with Kolmogorov–Smirnov non-parametric test.

TEER measurements do not account for pore size and can be influenced by temperature, media composition, cell maturation, and electrode placement [30, 31]. To validate the TEER results and further determine if the observed reductions in barrier function with U18666A and MβCD were size-selective, we performed permeability assays with different sized molecular weight tracers. Clearance volume of the small tracer molecule sodium fluorescein (MW = 376 Da) from the apical into the basolateral compartment increased with U18666A treatment. The sodium fluorescein permeability coefficient was approximately 9-fold higher in U18666A-treated (*p* < 0.0001) and nearly 20-fold higher in MβCD-treated (*p* < 0.0001) hiBMECs compared to untreated cells (Fig. 2A). However, the permeability effects of cholesterol depletion were smaller with larger molecular weight molecules. The permeability coefficient for 4 kDa dextran trended higher in U18666A-treated cells and was only ~ 5 times higher in MβCD-treated hiBMEC (*p* = 0.0159; Fig. 3B). There were no significant differences in the permeability coefficients for 10 kDa or 70 kDa dextran with either U18666A or MβCD (Fig. 3C, D). Thus, membrane cholesterol depletion increased hiBMEC permeability to small molecules only.

### hiBMEC Cholesterol Depletion Decreased Tight Junction Continuity

Since cell–cell tight junctions are critical to hiBMEC barrier function, we next determined how cholesterol depletion impacted hiBMEC tight junction proteins ZO-1, occludin, and claudin-5 continuity at the cell membrane. ZO-1, which links the actin cytoskeleton to other tight junction proteins [32], did not change continuity with U18666A treatment (Fig. 3A, B). However, both occludin and claudin-5 decreased continuity. Continuity of occludin, a tight junction protein vital for TJ stabilization [33], was ~ 13% lower in U18666A-treated cells (*p* < 0.0001). Continuity of claudin-5, a tight junction protein that regulates small molecule permeability [34, 35], was ~ 8% lower (*p* < 0.0001) in U18666A-treated cells.

### U18666A Reduced hiBMEC Claudin-5 Protein, Which was Prevented by HPβCD

**Fig. 4:**
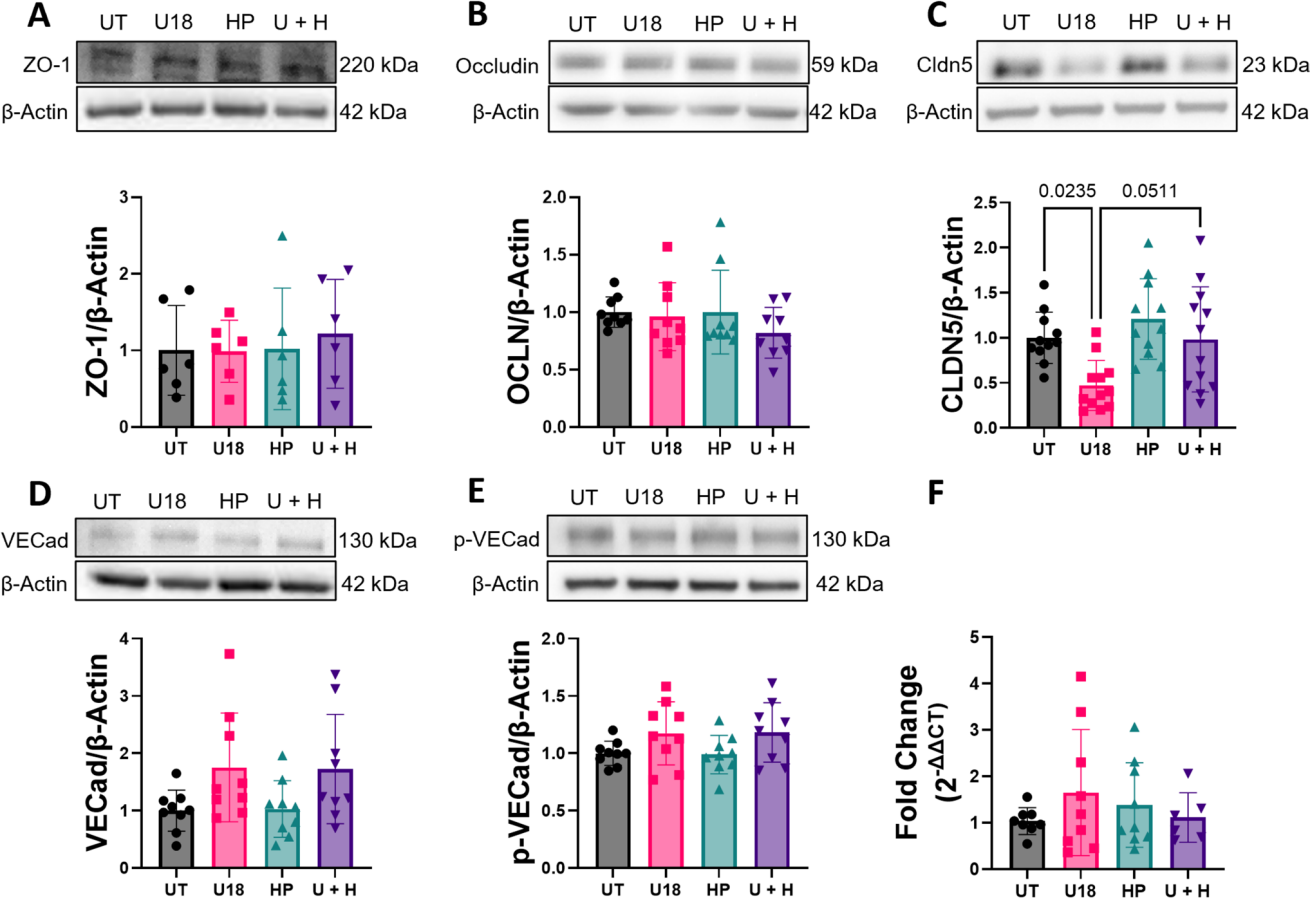
U18666A inhibition reduced claudin-5 protein, which was prevented by HPβCD co-treatment. Representative Western blots with quantification of hiBMEC that were untreated (UT) or treated with 10 μM U18666A (U18), 1000 μM HPβCD (HP), or both (U + H) for 48 h for **A** ZO-1 (*n* = 6 from two independent experiments) **B** occludin (*n* = 9, from 3 independent experiments) **C** claudin-5 (*n* = 11–12 from 4 independent experiments), **D** VE-cadherin (*n* = 9 from 3 independent experiments) and **E** phosphorylated VE-cadherin (*n* = 9 from 3 independent experiments). **F** CLDN5 expression, normalized to the housekeeping gene RLPL0 using the comparative CT method. (*n* = 6–9 from 3 independent experiments). Data shown are mean + /− SD. Statistical significance determined with Kruskal–Wallis non-parametric test followed by Dunn’s multiple comparison test.

To determine if decreased barrier integrity and tight junction continuity in U18666A-treated hiBMEC were due to changes in tight junction protein quantity, we measured tight junction proteins in U18666A-treated hiBMEC via Western blot. We also treated hiBMEC with HPβCD, which has been shown to reverse cholesterol accumulation, prolong animal life span, and slow human disease progression in NPC1 [17, 25, 36–38]. U18666A treatment did not change ZO-1 or occludin protein levels (Fig. 4A, B). U18666A also did not change total or phosphorylated VE-cadherin, an adherens junction protein that increases vascular permeability when it is phosphorylated (Fig. 4D, E) [39, 40]. However, U18666A treatment decreased hiBMEC claudin-5 by more than 50% (*p* = 0.0235; Fig. 4C). HPβCD alone did not impact tight junction protein quantity; however, when hiBMEC were treated with U18666A and HPβCD, claudin-5 was nearly maintained at baseline levels. *CLDN5* mRNA did not change significantly with either U18666A or HPβCD (Fig. 4F), indicating that the decrease in claudin-5 protein with U18666A may have been due to post-transcriptional mechanisms such as increased protein degradation, altered translation efficiency, or impaired trafficking. Thus, U18666A-induced cholesterol depletion decreased claudin-5 protein levels but did not impact occludin, VE-cadherin, or ZO-1.

### HPβCD Maintained hiBMEC Barrier Integrity and Continuous Tight Junctions

**Fig. 5:**
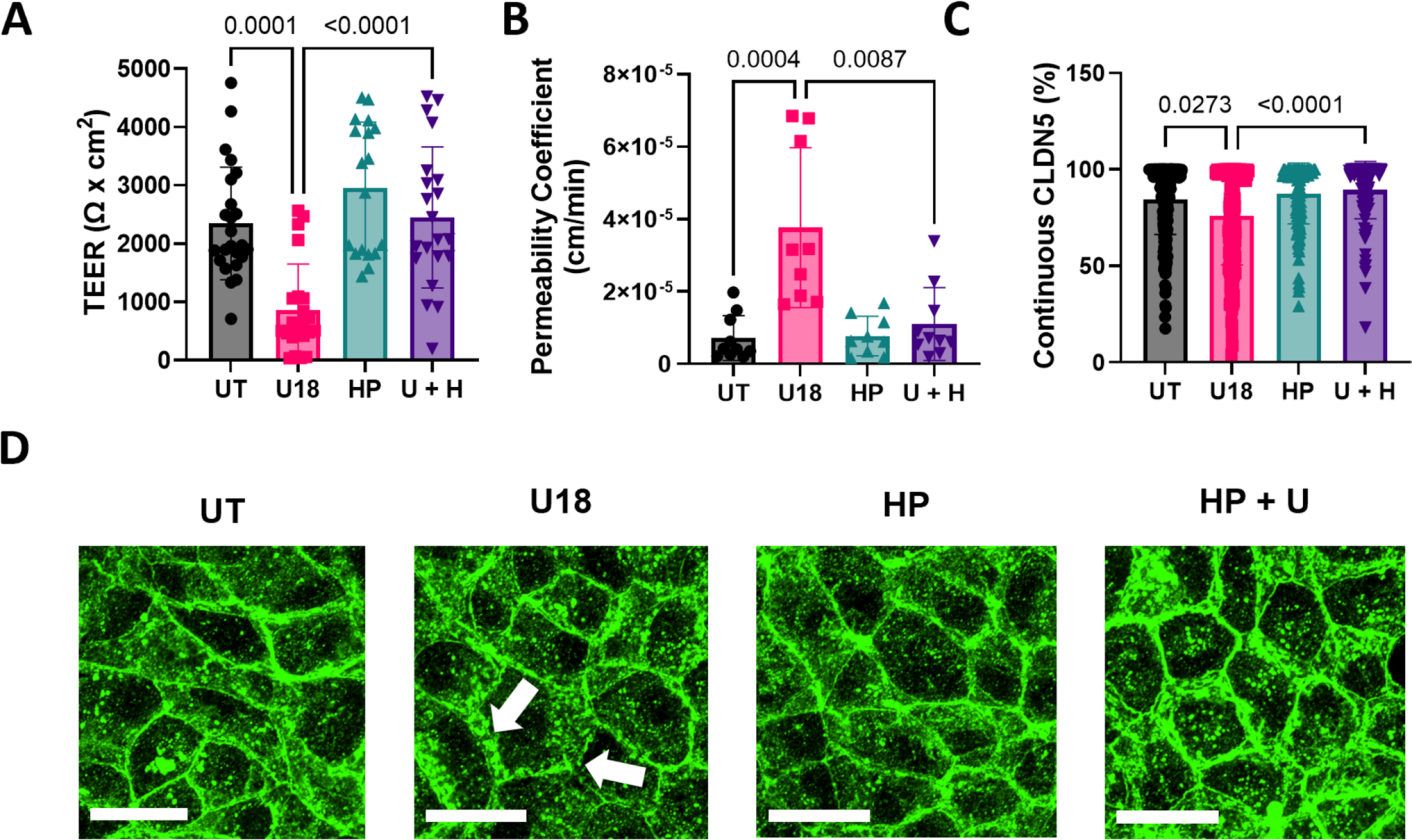
HPβCD prevented loss of barrier function and decrease in claudin-5 continuity in U18666A-treated hiBMEC. **A** TEER (*n* = 11–17 from 4 independent experiments) and **B** sodium fluorescein permeability coefficient (*n* = 10 from 2 independent experiments) at the end of each permeability experiment for hiBMEC that were untreated (UT) or treated with 10 μM U18666A (U18), 1000 μM HPβCD (HP), or both (U + H) for 48 h. **C** Percent of continuous claudin-5 (CLDN, *n* = 48–73 cells) as quantified by JaNAP and **D** representative claudin-5 (green) confocal microscopy images (Scale bar = 25 μM). White arrows highlight claudin-5 discontinuity. Data shown are mean + /- SD. Statistical significance determined with Kruskal–Wallis non-parametric test followed by Dunn’s multiple comparison test.

We then examined whether HPβCD could prevent changes in barrier function and tight junction protein continuity in U18666A-treated hiBMECs. HPβCD alone did not change barrier integrity. However, hiBMECs co-treated with U18666A and HPβCD for 48 h had higher TEER (*p* < 0.0001, Fig. 5A), sodium fluorescein permeability (*p* = 0.0114, Fig. 5B), and claudin-5 continuity (*p* < 0.0001, Fig. 5C, D) compared to hiBMEC treated with U18666A alone and were overall similar to untreated hiBMEC.

HPβCD is thought to alleviate lysosomal cholesterol accumulation and restore plasma membrane cholesterol. To determine whether specifically replenishing plasma membrane cholesterol would prevent the U18666A-induced decrease in barrier integrity, we co-treated hiBMECs with water-soluble cholesterol, which increases plasma membrane cholesterol [41]. We found that co-treatment of hiBMECs with U18666A and cholesterol prevented loss of TEER compared to hiBMECs treated only with U18666A (Supplemental Fig. 1).

### Glycolysis Inhibition Prevented Loss of Barrier Integrity in U18666A-Treated hiBMECs

**Fig. 6:**
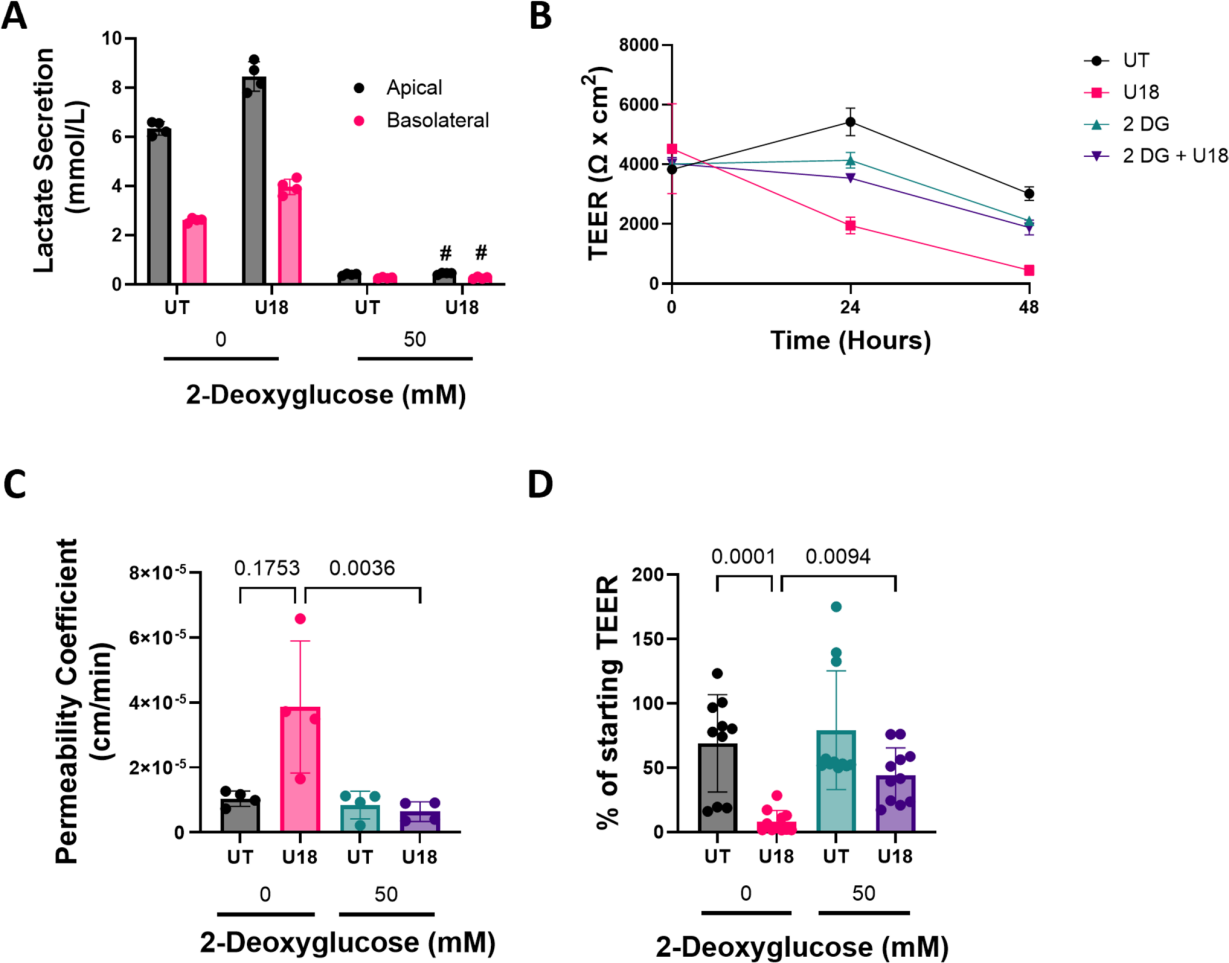
Glycolysis inhibition partially prevented U18666A-induced decrease in barrier integrity. **A** Apical and basolateral lactate concentration from hiBMECs cultured on Transwell inserts after 48 h of treatment with 10 μM U18666A with 0 or 50 mM 2-deoxy-D-glucose (2-DG). (*n* = 4 from one representative experiment of three independent experiments). # indicates significant reduction in 50 mM 2-DG conditions. **B** TEER measured over 48 h in hiBMECs treated with U18666A and 2-DG. (*n* = 4 from one representative experiment of three independent experiments). **C** Percent change in TEER for hiBMEC treated with U18666A and 2-DG. Percent change in TEER is defined as the final TEER value normalized to the initial TEER value for each well (*n* = 10–11 from three independent experiments). **D** Permeability coefficient for hiBMECs treated with U18666A and 2-DG. (*n* = 4 from one experiment). All data shown as mean + /− SD. Statistical significance determined with Kruskal–Wallis non-parametric test followed by Dunn’s multiple comparison test.

We previously showed that U18666A-treated hiBMEC had increased glycolytic activity, and increased glycolysis is known to increase cell permeability [17, 20]. We therefore questioned whether increased glycolysis was linked to decreased barrier function in U18666A-treated hiBMECs, particularly since we previously demonstrated that metabolic changes induced by U18666A are reversed by HPβCD [17]. hiBMECs treated with U18666A secreted more lactate both apically and basolaterally, consistent with increased glycolysis. When hiBMEC were treated for 48 h with 50 mM 2-deoxyglucose (2-DG), a glucose analog that inhibits the rate-limiting glycolytic enzyme hexokinase, lactate secretion dropped to nearly zero, indicating effective glycolytic inhibition (Fig. 6A). While U18666A decreased TEER more than 90% (Fig. 6B, C) and increased sodium fluorescein permeability coefficient 3.7-fold (Fig. 6D), 2-DG co-treatment resulted in a permeability coefficient (*p* = 0.0036) that was statistically similar to untreated cells and partially prevented decline in TEER (*p* = 0.0094). Therefore, glycolysis inhibition attenuated hiBMEC barrier disruption caused by U18666A treatment.

## Discussion

The BBB protects the brain from circulating neurotoxins and inflammatory compounds that contribute to neuropathology [42]. Despite the importance of cholesterol in barrier integrity, the BBB remains largely uncharacterized in diseases of cellular cholesterol dysregulation such as NP-C1. We now show that both NPC1 inhibition via U18666A and direct cholesterol depletion via MβCD decreased BMEC barrier integrity, increased BMEC permeability to small molecules, and disrupted claudin-5 continuity. Co-treatment with the potential NP-C1 therapeutic HPβCD prevented barrier integrity dysfunction. Together, these data suggest that BBB dysfunction may contribute to the neuropathogenesis of NP-C1 and potentially other inherited disorders of cholesterol metabolism, such as Smith-Lemli-Opitz Syndrome [43].

Cholesterol depletion via U18666A primarily affected the tight junction proteins claudin-5 and occludin. We observed decreased claudin-5 junction continuity and protein without a change in mRNA, which implicates an increase in claudin-5 degradation. Claudin-5 has been reported to undergo S-palmitoylation, a process in which the 16-carbon palmitic acid is covalently attached to the cysteine residue through a thioester bond [44]. Computational analysis suggests that palmitoylation stabilizes the interaction between claudin-5 and cholesterol, which shifts claudin-5 localization toward lipid rafts enriched in cholesterol [44]. In cholesterol-depleted BMEC, claudin-5 may get displaced to the cytoplasm, where it is then tagged for degradation. Indeed, previous work in epithelial cells demonstrated that cholesterol depletion with MβCD displaced claudins from lipid rafts [10, 11] and cell–cell junctions [12]. *In vivo* studies in rats also showed that cholesterol depletion decreased claudin-5 and increased BBB permeability [13].

Occludin membrane continuity also decreased with cholesterol depletion; however, we did not observe any changes in occludin protein levels. Occludin has been reported to be displaced out of lipid rafts following cholesterol depletion in Caco-2 epithelial cells [10, 11]. A later study showed that occludin displacement following cholesterol depletion in MDCK cells led to increased degradation by metalloproteinases [45]. However, the lack of change in occludin protein in our study suggests that the change in occludin continuity may instead be due to conformational changes and reduced interactions with the tight junction complex. Further work is needed to understand the specific nature of the observed differences in occludin morphology

Both claudin-5 and occludin are crucial for size-selective BMEC permeability. Claudin-5 regulates small molecule (< 800 Da) permeability. As a result, claudin-5 knockout mice showed increased permeability to Gd-DPTA (742 Da) and Hoescht (562 Da) but not microperoxidase (1.9 kDa), 4 kDa dextran, 10 kDa dextran, or serum albumin (68 kDa) [35, 46]. Claudin-5 and occludin together regulate BMEC permeability to molecules < 5 kDa. Knockout of both claudin-5 and occludin in mice increased BBB permeability to 3 kDa but not 10 kDa tracers, while knockout of either claudin-5 or occludin did not increase BBB permeability to either tracer [47]. Similarly, siRNA-knockdown of claudin-5 and occludin in mouse BMEC cultured on Transwell inserts increased permeability to a 4.3 kDa Aβ amyloid monomer but not an 8.7 kDa Aβ amyloid dimer [47]. Thus, the observed permeability increase to tracers with molecular weights between 376 and 4,000 Daltons was likely due to loss of both claudin-5 and occludin*.*

Interestingly, previous research showed that neither peripheral immune cells nor Evans blue dye infiltrated the brains of NPC1 mice [48]. These results agree with our data showing that cholesterol depletion does not increase BBB permeability to large objects such as immune cells. Evans blue dye, on the other hand, is a small molecule (< 1000 Da) that would be expected to permeate the brain in cholesterol-depleted conditions. However, Evans blue dye binds strongly to albumin, forming a 69 kDa molecule that is too large to cross the cholesterol-depleted BBB [49]. Together, these results suggest that BBB permeability should be further evaluated in NPC1 mice with small tracer molecules that do not bind to albumin, such as sodium fluorescein.

In addition to direct effects on claudin and occludin, cholesterol may affect tight junction formation through its impact on plasma membrane and cytoskeleton mechanics. Cholesterol is a key component of both caveolae and lipid rafts, which are plasma membrane microdomains where actin cytoskeleton interactions occur [50, 51]. Cholesterol depletion increased plasma membrane rigidity and decreased plasma membrane fluidity, which decreased lateral mobility of membrane proteins due to reorganization of the actin cytoskeleton [52–55]. Cholesterol depletion is postulated to impact the actin cytoskeleton through a reduction in the plasma membrane phospholipid phosphatidylinositol 4,5-bisphosphate (PIP2) [56, 57]. PIP2 directly interacts with actin, and its bioavailability mediates actin stability and actin–membrane interactions [57–59]. ZO-1 binds directly to the actin cytoskeleton and also links occludin to the actin cytoskeleton, and these interactions are critical for tight junction assembly and barrier function [60, 61]. Therefore, cholesterol depletion and subsequent perturbation of actin interactions with ZO-1 could also decrease tight junction formation and barrier function.

Barrier dysfunction in NPC1-inhibited BMECs may also partially be due to altered metabolic phenotype, as indicated by our experiments with 2-DG. Previous work in our lab demonstrated that NPC1 inhibition with U18666A increased hiBMEC glycolytic metabolism [17]. Enhanced glycolysis has been reported to decrease barrier integrity in endothelial cells. For example, a histamine-induced increase in glycolysis led to barrier disruption in human pulmonary microvascular endothelial cells. The glycolytic inhibitor 3-(3-pyridinyl)-1-(4-pyridinyl)-2-propen-1-one (3PO) prevented disruption of histamine-induced endothelial barrier disruption both *in vitro* and *in vivo* [20]. Glycolysis also generates highly reactive methylglyoxal (MGO) as a byproduct [62]. Co-treatment of BMECs with aminoguanidine, an MGO scavenger, prevented increases in endothelial permeability [63]. Glycolytic dysfunction could also disrupt barrier function through acidic stress induced by elevated lactate. Lactate increased vascular permeability in human umbilical vein endothelial cells by reducing VE-cadherin, ZO-1, and claudin-5 protein levels [64, 65]. Beyond glycolysis, mitochondrial ROS generation during oxidative phosphorylation can disrupt barrier integrity via occludin phosphorylation and dissociation from ZO-1 [66]. However, mitochondrial antioxidants did not restore BMEC barrier function in our cells (data not shown). Together, our data suggest that elevated glycolysis plays a role in tight junction disruption and barrier dysfunction of U18666A-treated hiBMECs.

HPβCD has been studied as a treatment for NP-C1, since it releases cholesterol from the lysosome and facilitates its transport into the plasma membrane and other organelles [25, 67]. We previously observed that HPβCD maintained membrane cholesterol and partially rescued metabolic changes in U18666A-treated hiBMECs [17]. We now show that the HPβCD therapeutic benefit extends to barrier function as well. This could be an important consideration for therapeutic protocols, as barrier function restoration with HPβCD may paradoxically prevent subsequent therapeutic delivery to the brain in NP-C1. Thus, later HPβCD doses may be less effective than the early doses as BBB integrity is restored.

Although this study shows the impact of cholesterol depletion on barrier function, there are a few limitations to consider. Some U18666A effects may arise due to off-target effects, including U18666A intercalation into the cell membrane and inhibition of dehydrocholesterol reductase (DHCR24), which converts desmosterol into cholesterol [68]. Although in our prior work, we did not observe an increase in desmosterol in U18666A-treated hiBMECs [17], we do not know if membrane intercalation affected U18666A-treated hiBMECs. In addition, we used a simplified BBB model that only included BMECs. Other BBB cells, such as astrocytes and neurons, promote BBB integrity and elevate TEER when co-cultured with BMECs [69]. The other cell types may provide supportive cues that mitigate BBB dysfunction in NP-C1. Furthermore, our study shows that HPβCD can prevent loss of barrier integrity in NPC1-inhibited hiBMECs but does not show whether it can restoring barrier integrity in hiBMECs that are already NPC1 deficient. These studies will be imperative to perform in the future, particularly in genetic knockout models which more accurately recapitulate the NP-C1 phenotype.

This study shows that cholesterol depletion disrupts hiBMEC claudin-5 and occludin, increasing hiBMEC permeability to small molecules (< 4 kDa). Elevated BBB permeability to small molecules may disrupt the brain by increasing penetrance of neurotoxic metabolites, metals, and other compounds throughout a patient’s lifespan. However, we could also take advantage of size-specific permeability changes to improve brain therapeutic delivery by constraining the therapeutics to small molecular weights. Cholesterol-induced BBB permeability should be studied in animal models and individuals with NP-C1 to characterize the extent of BBB dysfunction *in vivo*. These studies would substantiate the role of the BBB in contributing to or even precipitating neurological manifestations of NP-C1 and highlight the brain endothelium as a potential therapeutic target to slow disease progression.

## Supplementary Information

Below is the link to the electronic supplementary material.

Supplementary file1 (DOCX 42 kb)

## Data Availability

All data are available from the corresponding author (AMC) upon reasonable request.
