# Supplementary material for: Cholesterol Depletion with U18666A and Methyl-β Cyclodextrin Increased Small Molecule Permeability Across Brain Microvascular Endothelial Cells
Source: Ann Biomed Eng. 2025 Sep 17;53(11):3222–36. doi: 10.1007/s10439-025-03841-9 (PMC12575569; doi:10.1007/s10439-025-03841-9)
Supplement: Supplementary file 1 — Supplementary file1 (DOCX 42 kb) [file 10439_2025_3841_MOESM1_ESM.docx]

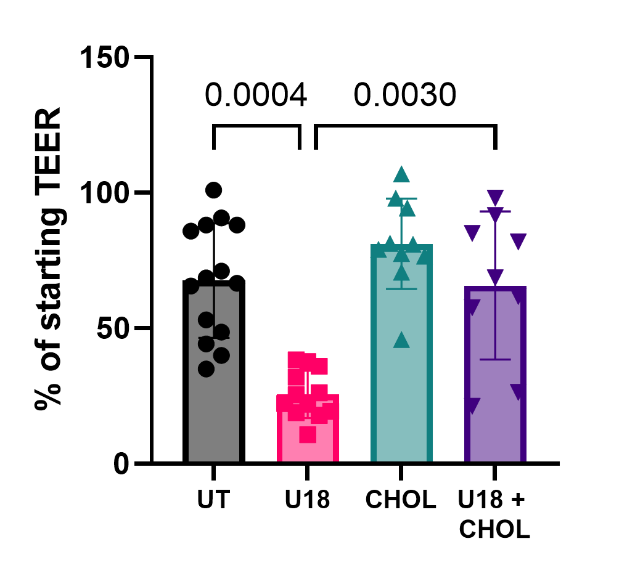


**Supplemental Figure 1. Water-soluble cholesterol (CHOL) prevented loss of barrier function in U18666A-treated hiBMEC.** TEER for hiBMEC that were untreated (UT) or treated with 10 μM U18666A (U18), 50 μM water-soluble cholesterol (CHOL), or both (U18 + CHOL) for 48 hours. n = 9-14 from 3 independent experiments. Statistical significance determined with Kruskal-Willis non-parametric test followed by Dunn’s multiple comparison test.
